# Brazilian Journal of Cardiovascular Surgery 30 years: Does the Impact Factor Matter?

**DOI:** 10.5935/1678-9741.20160050

**Published:** 2016

**Authors:** Paulo Roberto B. Evora

**Affiliations:** 1 Full Professor, Department of Surgery and Anatomy, Faculdade de Medicina de Ribeirão Preto da Universidade de São Paulo (FMRP-USP), Ribeirão Preto, SP, Brazil

"Brazil is much greater than the crises it faces. It is essential to show what we are capable of in order to stand amidst other nations". (Domingo M. Braile, 2016)

Domingo Braile [Brazilian Journal of Cardiovascular (BJCVS) Editor] wrote: "We have a constant obsession with having a better impact factor and, as a result, a Qualis ranking compatible with our specialty, will suffer another drawback: due to the change of name, the BJCVS citation will be only in English and thereby counted by Thomson and Scopus from now on. These are problems to be faced in order to improve for the future"^[1]^. I think that our 0.526 impact factor, despite all our efforts, it is not compatible with the BJCVS Thomson Reuters evaluation. Maybe this is the only reason for sadness during the BJCVS 30 years celebration. However, some considerations may alleviate this feeling.

Randy Schekman, a US biologist who won the 2013 Nobel prize in physiology or medicine receiving his prize in Stockholm, said his lab would no longer send research papers to the toptier journals, Nature, Cell and Science. Schekman said pressure to publish in "luxury" journals encouraged researchers to cut corners and pursue trendy fields of science instead of doing more important work. The problem was exacerbated, he said, by editors who were not active scientists, but professionals who favoured studies that were likely to make a splash. Writing in the Guardian, Schekman raises serious concerns over the journals' practices and calls on others in the scientific community to take action. "I have published in the big brands, including papers that won me a Nobel prize". But no longer, he writes. "Just as Wall Street needs to break the hold of bonus culture, so science must break the tyranny of the luxury journals." A journal's impact factor is a measure of how often its papers are cited, and is used as a proxy for quality. But Schekman said it was "toxic influence" on science that "introduced a distortion". He writes: "A paper can become highly cited because it is good science - or because it is eye-catching, provocative, or wrong"^[2]^.

Although Dr. Sheckman's piece has received a fair share of criticism, including remarks of evident hypocrisy, it does bring attention to certain emerging problems within the scientific community. One major concern, which Sheckman touched upon, is the widespread use of the impact factor as a measure of research quality and productivity. Derived from citations to all articles in a specific journal, the impact factor was originally established by Thomson Reuters Corporation as a tool to help librarians identify journals to purchase^[3]^. However, when applied by funding agencies, academic institutions, and other parties, the impact factor is often used as the primary parameter to evaluate an individual's or an institution's scientific contributions. Meanwhile, scientists alike agree that the impact factor does not appropriately measure the quality of a scientific article nor does it reflect how influential the work is in the field^[3]^. Thus, in recognizing the misleading influence of the impact factor, scientists across various disciplines are voicing a call for change.

Finally, it would be appropriate to highlight some points that are involved directly and indirectly with the impact factor: a) High taxes for publications; b) The so-called "research consortium" with papers that have up to 200 co-authors; c) Often abusive self-citation rates, and; d) The billionaires news editorial groups mergers etc. For our society the impact factor does not matter. So, Dr. Braile we do not have to ask "for whom the bell tolls, we are not an island, the bell tolls for our BJCVS 30 years" (Jon Donne)... CONGRATULATIONS...
